# Peer Review of “The Influence of SARS-CoV-2 Variants on National Case-Fatality Rates: Correlation and Validation Study”

**DOI:** 10.2196/38548

**Published:** 2022-05-24

**Authors:** Manoja K Das

**Affiliations:** 1 Director Projects The INCLEN Trust International New Delhi India

**Keywords:** SARS-CoV-2, COVID-19, variants of concern, case-fatality rates, virulence, vaccine effectiveness, correlation study

*This is a peer-review report submitted for the paper “The Influence of SARS-CoV-2 Variants on National Case-Fatality Rates: Correlation and Validation Study.”*

## Round 1 Review

### General Comments

This paper [1] presents the changes in the case-fatality rate (CFR) due to COVID-19 variants in different countries.

### Specific Comments

#### Major Comments

##### 1. Abstract

1.1. Should include a conclusion section

1.2. Results: A summary of the results in terms of variation in CFR according to the variants needs to be mentioned.

##### Main Manuscript

###### 2. Objective

2.1. Specify the year for November 1

2.2. Figure 2: What do the different shades indicate? It should be clarified in the footnote. November spelling.

###### 3. Methods of Analysis

3.1. Data sources should be specified for the different countries. The analysis should also mention the methods used for data analysis and presentation in the tables. The data on the infected case load should be used along with the CFR/proxy CFR (pCFR).

3.2. pCFR: Full form when used first. The proxy CFR or pCFR should be used consistently in the text.

###### 4. Results

4.1. Figure 7: What was the source of the data for the cofactors in these countries? It should be specified.

4.2. Correlation between regional CFRs: The pairing of the countries should be mentioned in the Methods. Which statistical test was used for this correlation analysis? This should be mentioned in the Methods.

###### 5. Discussions and Conclusions

Discussion and Conclusion should be separated.

## Abbreviations

CFR: case-fatality rate
pCFR: proxy case-fatality rate
